# Airway Epithelial NF-κB Activation Promotes *Mycoplasma pneumoniae* Clearance in Mice

**DOI:** 10.1371/journal.pone.0052969

**Published:** 2012-12-28

**Authors:** Di Jiang, Mark L. Nelson, Fabienne Gally, Sean Smith, Qun Wu, Maisha Minor, Stephanie Case, Jyoti Thaikoottathil, Hong Wei Chu

**Affiliations:** 1 Department of Medicine, National Jewish Health and the University of Colorado Denver, Denver, Colorado, United States of America; 2 Department of Immunology, National Jewish Health and the University of Colorado Denver, Denver, Colorado, United States of America; 3 Business and Science Development, Echelon Biosciences Inc., Salt Lake City, Utah, United States of America; National Institute of Environmental Health Sciences, United States of America

## Abstract

**Background/Objective:**

Respiratory infections including atypical bacteria *Mycoplasma pneumoniae* (Mp) contribute to the pathobiology of asthma and chronic obstructive pulmonary disease (COPD). Mp infection mainly targets airway epithelium and activates various signaling pathways such as nuclear factor κB (NF-κB). We have shown that short palate, lung, and nasal epithelium clone 1 (SPLUNC1) serves as a novel host defense protein and is up-regulated upon Mp infection through NF-κB activation in cultured human and mouse primary airway epithelial cells. However, the *in vivo* role of airway epithelial NF-κB activation in host defense against Mp infection has not been investigated. In the current study, we investigated the effects of *in vivo* airway epithelial NF-κB activation on lung Mp clearance and its association with airway epithelial SPLUNC1 expression.

**Methodology/Main Results:**

Non-antimicrobial tetracycline analog 9-t-butyl doxycycline (9-TB) was initially optimized in mouse primary tracheal epithelial cell culture, and then utilized to induce *in vivo* airway epithelial specific NF-κB activation in conditional NF-κB transgenic mice (CC10-_CA_IKKβ) with or without Mp infection. Lung Mp load and inflammation were evaluated, and airway epithelial SPLUNC1 protein was examined by immunohistochemistry. We found that 9-TB treatment in NF-κB transgene positive (Tg+), but not transgene negative (Tg−) mice significantly reduced lung Mp load. Moreover, 9-TB increased airway epithelial SPLUNC1 protein expression in NF-κB Tg+ mice.

**Conclusion:**

By using the non-antimicrobial 9-TB, our study demonstrates that *in vivo* airway epithelial NF-κB activation promotes lung bacterial clearance, which is accompanied by increased epithelial SPLUNC1 expression.

## Introduction

Bacterial infection is involved in the pathogenesis of asthma and chronic obstructive pulmonary diseases (COPD), two of the most common respiratory diseases worldwide. Several strains of bacteria were identified in the airways of asthma and COPD patients, including *nontypeable Haemophilus influenza*, *Moraxella catarrhalis* and atypical bacteria such as *Mycoplasma pneumoniae* (Mp) [1]. Mp, for instance, has been associated with the exacerbations as well as the persistence of asthma and COPD [2], [3]. Treatment of Mp infection is challenging, as most antibiotics are bacteriostatic, but not bactericidal for Mp [4]. Therefore, understanding the host defense mechanisms against Mp infection would offer more effective therapies to treat chronic lung diseases.

Mp infection is known to predominantly target airway epithelium, leading to epithelial damage and inflammatory cytokine production. Airway epithelium, the first line of host defense against environmental hazards, utilizes various signaling pathways to modulate host defense against bacteria [5], [6], [7]. For example, airway epithelial nuclear transcription factor κB (NF-κB) can be activated following Mp infection [5], which promotes the production of chemokines involved in leukocyte recruitment and activation. Thus, studying the role of NF-κB in airway epithelial cell responses to bacterial infection is critical to find better strategies to eliminate bacteria from airways of asthma and COPD patients.

Several groups of investigators have generated doxycycline (Dox)-inducible NF-κB transgenic mice to study the role of airway epithelial NF-κB activation in airway allergic inflammation [8], [9]. So far, the role of airway epithelial NF-κB signaling in lung bacterial infection and clearance remains poorly understood. Although Chen et al has demonstrated the feasibility of *Pseudomonas aeruginosa* (Pa) infection in Dox-inducible NF-κB transgenic mice [10], that study is limited for its broad application because Pa is resistant to Dox [11]. Indeed, Pa is about 266 times more resistant to the bactericidal effect of Dox than other strains of bacteria (*e.g.* Mp) that are highly relevant to some of the most prominent lung diseases including asthma and COPD [12], [13], [14]. To overcome the antimicrobial activity of Dox, in the present study, we utilized non-antimicrobial tetracycline analog tetracycline analog 9-t-butyl doxycycline (9-TB) in conditional NF-κB transgenic mice that were infected with Mp. 9-TB is a novel tetracycline analog that has been used in cell culture and animal studies [15], [16]. The primary goal of our study was to test if *in vivo* airway epithelial NF-κB activation was critical to lung defense against Mp.

Our secondary goal is to reveal the potential mechanisms by which *in vivo* airway epithelial NF-κB activation enhances host defense against Mp. Our previous publications have shown that short palate, lung, and nasal epithelium clone 1 (SPLUNC1), a member of the PLUNC family that is localized in large airway epithelium, exerts antimicrobial activity against Mp. Moreover, SPLUNC1 was induced in cultured human and mouse primary airway epithelial cells upon Mp infection largely through the activation of NF-κB pathway [5], [17], [18]. Therefore, in the present study, we examined mouse (*in vivo*) airway epithelial SPLUNC1 expression following NF-κB activation to provide a potential mechanism for NF-κB-mediated host defense against bacterial infection.

## Results

### Validation of Non-antimicrobial Feature of Tetracycline Analog 9-t-butyl Doxycycline (9-TB)

**Figure 1:**
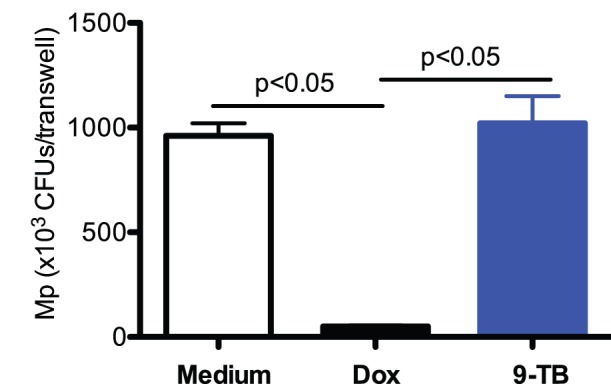
Validation of the non-antimicrobial feature of a tetracycline analog 9-t-butyl doxycycline (9-TB). Tracheal epithelial cells from wild-type C57BL/6 mice were isolated and cultured under air-liquid interface (ALI) condition as described in the Materials and Methods section. The effects of medium control, doxycycline (Dox, 0.5 µg/ml) or 9-TB (0.5 µg/ml) on Mp growth in the apical supernatants of epithelial cells were examined at 24 hour post infection. N = 3; CFUs = colony forming units. Data are expressed as means ± SEM.

To date, *in vivo* bacterial studies in Dox-induced NF-κB transgenic mouse models were impossible because of the broad spectrum of antimicrobial activity of Dox. Thus, we determine if 9-TB exerted any antimicrobial activity in mouse tracheal epithelial cell air-liquid interface (ALI) cultures with Mp infection. 24 hours post infection, Dox treatment markedly reduced Mp load compared to the control medium, while 9-TB at both 0.5 and 2 µg/ml did not show antimicrobial activity against Mp. Figure 1 demonstrates the effects of 9-TB at 0.5 µg/ml on Mp load.

### Lung NF-κB Activation in 9-TB-treated CC10-_CA_IKKβ Tg+ (NF-κB Tg+) Mice

**Figure 2:**
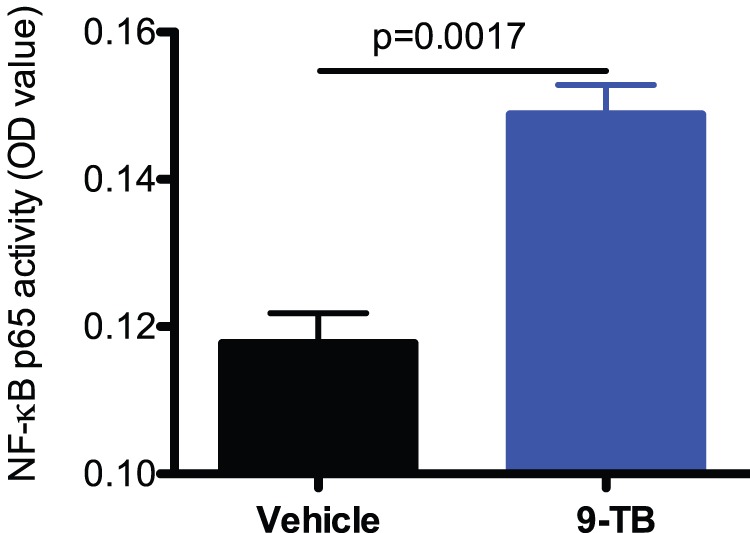
9-TB enhances NF-κB activation in saline-treated CC10-_CA_IKKβ Tg+ mice. NF-κB activity in 9-TB- and saline-treated CC10-_CA_IKKβ Tg+ mice was measured by using the NF-κB p65 ELISA in nuclear proteins extracted from mouse lungs (n = 4 mice per group). Data are expressed as means ± SEM.

To address if 9-TB increases NF-κB activation, we measured whole lung NF-κB activation levels in CC10-_CA_IKKβ Tg+ mice with or without administration of 9-TB (please refer to “Material and Method” section for details on *CC10-_CA_IKKβ transgenic mouse strain*). At 24 hr after the last 9-TB treatment, 9-TB treated-mice, as compared to vehicle control mice, demonstrated increased NF-κB activation (Figure 2).

### Increased Lung Leukocytes and Cytokines in 9-TB-treated NF-κB Tg+ Mice

**Figure 3:**
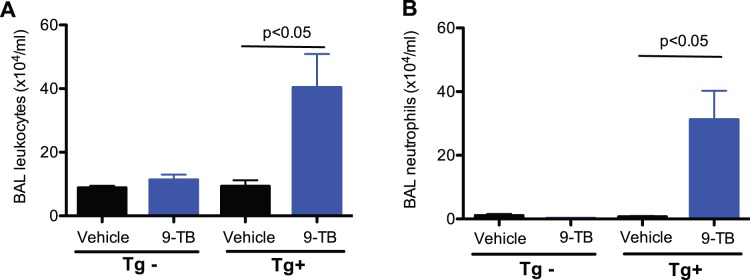
9-TB treatment increases leukocytes in bronchoalveolar lavage (BAL) fluid of CC10-_CA_IKKβ Tg+ mice with saline treatment. (**A**) – total leukocytes; (**B**) – neutrophils. N = 4–6 mice per group. Data are expressed as means ± SEM.

**Figure 4:**
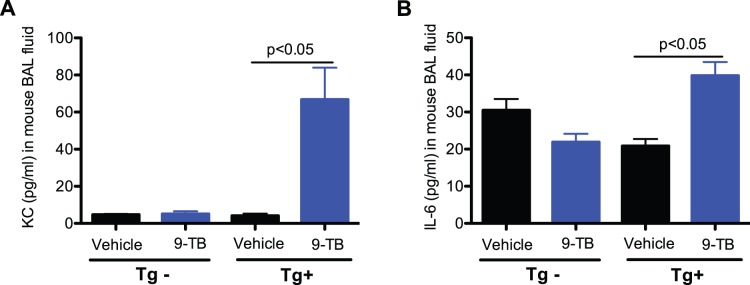
9-TB treatment increases KC and IL-6 levels in CC10-_CA_IKKβ transgene positive (Tg+), but not transgene negative (Tg–) mice with saline treatment. (**A**) – KC; (**B**) – IL-6. N = 4–6 mice per group. Data are expressed as means ± SEM.

NF-κB activation regulates the production of chemokines and cytokines, resulting in leukocyte recruitment. Here, we evaluated lung leukocyte and pro-inflammatory cytokine levels to confirm the functional consequences of 9-TB-mediated airway epithelial NF-κB activation. Total leukocytes including neutrophils in bronchoalveolar lavage fluid (BALF) of 9-TB treated NF-κB Tg+ mice were significantly increased (Figure 3A and 3B). After 9-TB treatment, levels of chemokine KC (a homolog to human IL-8) and interleukin-6 *(*IL-6) were significantly elevated in BALF of NF-κB Tg+, but not NF-κB Tg– mice (Figures 4A and 4B).

### Reduced Lung Mp Load with Increased Airway Epithelial SPLUNC1 in 9-TB-treated NF-κB Tg+ Mice

**Figure 5:**
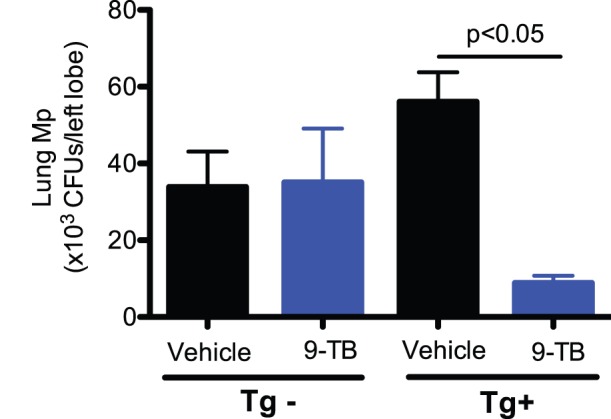
9-TB reduces lung Mp load in CC10-_CA_IKKβ transgene positive (Tg+) mice. Left lungs from Mp-infected mice (24 hours after infection) were homogenized and plated on PPLO-plates to count Mp CFUs. 9-TB significantly reduced lung Mp load in Tg+ mice, but had a minimal impact on Mp load in transgene negative (Tg–) mice. N = 4–6 mice per group. Data are expressed as means ± SEM.

Having shown that 9-TB was able to induce lung NF-κB activation, we then determined the effects of airway epithelial NF-κB activation on lung bacterial clearance. After 24 hrs of Mp infection, 9-TB pretreatment significantly reduced lung Mp load in NF-κB Tg*+* mice, but not in Tg− mice (Figure 5).

Although lung bacterial load was reduced in 9-TB-treated and Mp-infected Tg+ mice, the underlying molecular mechanism remains unclear. To explore the potential *in vivo* mechanisms of reduced bacterial load in 9-TB-treated and Mp-infected Tg+ mice, we performed immunohistochemistry to examine SPLUNC1 protein in mouse airway epithelial cells. Our previous publications have shown that: (1) SPLUNC1 is critical to lung Mp clearance because SPLUNC1 knockout mice had higher levels of lung bacterial load than the wild-type mice [17]; and (2) NF-κB activation following Mp infection was largely responsible for SPLUNC1 up-regulation in cultured mouse airway epithelial cells [5]. Within the NF-κB Tg*+* mice, 9-TB induced SPLUNC1 protein in airway epithelial cells as compared to vehicle solution (Figures 6A and 6B). Quantitatively, SPLUNC1 protein in airway epithelium was induced up to 6-fold in NF-κB Tg*+* mice (Figure 6C). Collectively, our data suggested that 9-TB-mediated airway NF-κB activation decreased lung Mp load coupled with increased airway epithelial SPLUNC1 protein expression.

**Figure:**
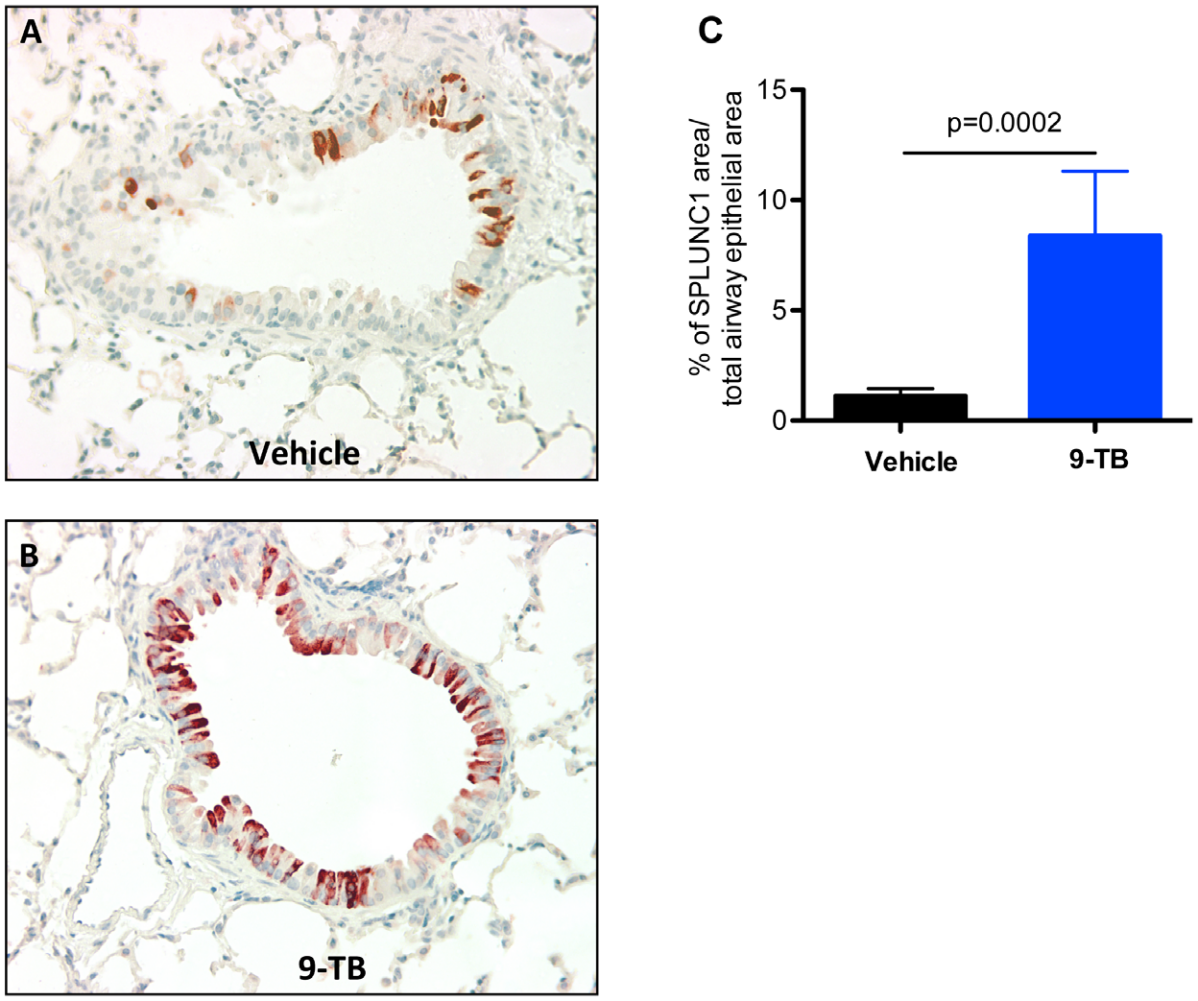
9-TB up-regulates airway epithelial SPLUNC1 protein in CC10-_CA_IKKβ transgene positive (Tg+) mice. Lungs from saline-treated Tg+ mice were processed for SPLUNC1 immunohistochemistry. Representative SPLUNC1 staining in medium-size airways of Tg+ mice treated with vehicle solution (**A**) and 9-TB (**B**). Quantitative data of airway SPLUNC1 protein (**C**) are expressed as a percentage of stained area versus total (stained plus non-stained) airway epithelial area. N = 4 mice per group. Data are expressed as means ± SEM.

## Discussion

Our current study has clarified the *in vivo* role of airway epithelial NF-κB activation in host defense against Mp infection by administrating a non-antimicrobial tetracycline analog 9-TB in conditional NF-κB transgenic mice. Specifically, our data suggest that airway epithelial cell NF-κB activation enhances Mp clearance form the lung.

One of the primary barriers in the study of *in vivo* airway epithelial NF-κB host defense functions is the lack of an appropriate animal model. Although Dox-inducible NF-κB transgenic mice have been generated to study the role of NF-κB activation in airway inflammation [8], [9], studying bacterial infection in these mice was almost impossible due to the broad-spectrum antibiotic nature of Dox. Therefore it is important to develop a novel animal model to study the role of airway epithelial NF-κB against bacteria that are very sensitive to Dox. The major strength of our current study is that we have overcome the broad bactericidal effect of Dox by using a non-antimicrobial tetracycline analog (i.e., 9-TB) to induce NF-κB activation in CC10-_CA_IKKβ (NF-κB transgenic) mice. Our mouse model clearly offers a novel approach to study how airway epithelial NF-κB activation promotes clearance of bacteria that are susceptible to Dox. Our data demonstrated that 9-TB significantly reduced lung Mp load in transgenic positive mice as compared with transgenic negative mice. Although we only included Mp in this study, we speculate that 9-TB can be used in CC10-_CA_IKKβ mice to study the role of airway epithelial NF-κB in lung infection with other strains of bacteria. Airway epithelial NF-κB activation in healthy human subjects may serve as a protective mechanism against bacterial infection. As we reported previously [19], bacteria-induced NF-κB activation under an allergic or Th2 cytokine (a major mediator in asthma lungs) milieu is dampened compared with that under a healthy condition, which may explain the persistent nature of bacterial infection in asthma.

Similar to previous studies showing lung NF-κB activation following Dox treatment in CC10-_CA_IKKβ mice [8], 9-TB also increased lung NF-κB activation. In addition, we found that NF-κB activation-associated inflammatory markers were also increased by 9-TB, including leukocytes (e.g., neutrophils) and cytokines (e.g., KC and IL-6). Our data further indicate that use of 9-TB is an excellent approach to activate airway epithelial NF-κB for studying the impact of *in vivo* NF-κB activation on various epithelial functions following bacterial infection.

Our previous study suggests that SPLUNC1 was inducible upon Mp infection in cultured airway epithelial cells largely through NF-κB pathway [5]. Our current study has extended our previous work by revealing *in vivo* airway epithelial SPLUNC1 up-regulation following NF-κB activation. Future studies are warranted to explicitly define the contribution of airway epithelial SPLUNC1 up-regulation to bacterial clearance in 9-TB-treated NF-κB transgenic mice. This could be achieved by applying a mouse SPLUNC1 neutralizing antibody prior to bacterial infection.

There are several limitations to our present study. First, it focused on an acute (e.g., day 1 post infection) infection model. Although our acute mouse model is highly relevant to acute exacerbations of lung diseases, chronic bacterial infection model will be needed in the future to study the role of airway epithelial NF-κB pathway in disease progression. Second, as NF-κB activation in mouse airway epithelium activates an array of host defence molecules (e.g., KC and IL-6), the enhanced airway epithelial SPLUNC1 expression in 9-TB-treated NF-κB transgenic mice is expected to serve only as one of the mechanisms involved in enhanced lung Mp clearance. To explicitly demonstrate the role of SPLUNC1 in airway epithelial cell NF-κB-mediated lung defense against Mp, future studies are warranted to breed SPLUNC1 knockout mice or their wild-type littermates with NF-κB transgenic mice, and infect the new strains of mice with Mp. Moreover, as other mediators (e.g., KC and IL-6) induced by NF-κB activation have been shown to promote Mp clearance [19], the contribution of those additional mediators will be considered in our future studies by using knockout mice or neutralizing antibodies. Addtionally, we may need to examine other antimicrobial substances (e.g., lactotransferrin and β defensin 2) that can also be increased following NF-κB activation. Third, although the canonial NF-kB pathway is predominatly activated in our CC10-_CA_IKKβ mouse model [20], [21], [22], IKKβ activation may have NF-κB-independent effects. For example, IKKβ activation can phosphorylate adaptor protein DOK1, and subsequently inhibit MAP kinase signaling [23]. Because MAP kinases are involved in inflammatory cytokine production, and even SPLUNC1 induction [24] during bacterial infection, it is likely that IKKβ activation may serve as a negative regulatory mechanism to prevent excessive activation of canonical NF-κB pathway. The balance of IKKβ-induced NF-κB activation and MAP kinase inhibition during mycoplasma infection warrants future studies to better understand the functions of IKKβ-mediated signaling in airway epithelial cells. Lastly, in the current study, we only evaluated the antimicrobial effect of 9-TB on Mp. Whether 9-TB has any antimicrobial activity against other strains of bacteria (e.g., *Streptococcus pneumoniae*, *E. coli*) remains to be determined in future studies.

In summary, the current study has significantly advanced our understanding regarding the *in vivo* role of airway epithelial NF-κB activation in lung Mp infection. Our unique research approach (e.g., use of 9-TB) will facilitate future investigations into the role of airway epithelial NF-κB in respiratory infections of other strains of bacteria that are relevant to various lung diseases such as asthma, COPD and cystic fibrosis.

## Materials and Methods

### Ethics Statement

Experimental animals used in this study were covered by a protocol approved by Institutional Animal Care and Use Committee (IACUC) of National Jewish Health. All experimental procedures were carried out to minimize animal discomfort, distress, and pain by following the American Veterinary Medical Association Guidelines.

### Animals

Mice with Dox-inducible constitutively active (CA) version of inhibitor of κB (IκB) kinase-beta (IKKβ) under transcriptional control of the rat CC10 promoter (CC10-_CA_IKKβ mice) were kindly provided by Dr. Yvonne M.W. Janssen-Heininger at University of Vermont (Burlington, VT). These transgenic mice have been previously validated for having the inducible transgene expression exclusively in the airway epithelium [20]. Both Tg+ and Tg− (wild-type) C57BL/6 mice (8–10 wk old) were inbred and housed in our biological resource center under pathogen-free conditions. All of the mice were tested to establish that they were virus and *M. pulmonis* free.

### Mp Preparation

Mp (strain FH, American Type Culture Collection 15531) was grown in SP-4 broth for 5 days at 37°C. The adherent Mp was harvested, spun and resuspended in PBS with 20% FBS, aliquoted and frozen in −80°C for infecting mouse tracheal epithelial cell culture, as well as mice in a consistent manner. Frozen Mp aliquots were thawed, spun and resuspended in SP-4 broth on the day of infection. After a 2 hour incubation at 37°C, Mp was spun at 6000 rpm for 5 minutes and resuspended in sterile saline to yield 1×10^8^ colony forming units (CFUs)/50 µl for infecting mice, or resuspended in cell culture medium to yield 1 CFU/cell for infecting cultured mouse primary tracheal epithelial cells.

### Culture of Mouse Primary Tracheal Epithelial Cells to Test the Non-antimicrobial Feature of 9-TB

We obtained 9-TB from Paratek Pharmaceuticals (Boston, MA) through a Material Transfer Agreement (MTA) for the current study. Details of 9-TB have been described in previous publications [15], [16]. 9-TB is commercially available from Mark Nelson at Echelon Biosciences Inc., Salt Lake City, Utah, USA.

Mouse tracheal epithelial cell isolation and air-liquid interface (ALI) culture were carried out as previously reported [25] to test whether 9-TB exerted the antimicrobial (e.g., mycoplasma) activity. Briefly, tracheas from the wild-type C57BL/6 mice were isolated, cut longitudinally and pooled for digestion with 0.1% protease. The released cells were seeded on collagen-coated polyester transwell inserts (12 well plate, 0.4 um pore size; Corning Costar, Corning, NY) at 4×10^4^ cells in 500 µl DMEM/BEBM (1∶1) with supplements [26]. Cells were in immersed culture for 7 days to reach 100% confluence, and then switched to ALI condition by reducing the apical medium to 50 µl. By utilizing ALI culture environment, the cells undergo mucociliary differentiation, thus mimicking *in vivo* airway epithelial cell biology. At day 10 of ALI culture, 9-TB at concentrations that were comparable to Dox treatment [27] were added to epithelial cells. In previous studies [28], [29], the minimal inhibitory concentration of Dox for Mp ranges from 0.01 to 1 µg/ml. Considering the complexity of mouse tracheal epithelial culture system versus the agar plate culture system used in previous studies, we chose doses of 0.5 and 2.0 µg/ml to test the antimicrobial activity of 9-TB as well as Dox (positive control). Briefly, medium control, 0.5 or 2 µg/ml of 9-TB or Dox was administered to both apical and basal sides of epithelial cells. All treatments were refreshed daily for two consecutive days, followed by Mp infection at 1 CFU/cell. Apical supernatants were collected at 24 hours post infection for Mp culture and quantification.

### Mouse Model of Mp Infection with Intraperitoneal (i.p.) Injection of 9-TB

As our data demonstrated non-antimicrobial feature of 9-TB, mice were administered 200 µl of 9-TB at 25 mg/kg (or 0.5 mg/20 g body weight) in vehicle solution (PBS solution containing 25 mM HCl) or vehicle control solution through i.p. injection once a day for 4 consecutive days. The dose selection of i.p. injection of 9-TB was based on previous Dox studies showing a dose range of 10 to 100 mg/kg body weight, and the lung tissue Dox concentration after an i.p. injection [30], [31], [32], [33]. Two hours after the last 9-TB treatment, mice were anesthetized via i.p. injection of tribromoethanol (Avertin; Sigma-Aldrich, St. Louis, MO) at 0.25 g/kg (or 5 mg/20 g body weight), and then inoculated intranasally with 50 µl Mp at 1×10^8^ CFU or 50 µl saline. Mice were sacrificed 24 hours later.

### Bronchoalveolar Lavage (BAL) and Lung Tissue Processing

NF-κB transgenic mouse lungs were lavaged with 1 ml of sterile saline. Cell-free BAL fluid were stored at –80°C for cytokine analysis. Cytospins of mouse BAL cells were stained with Diff-Quick Kit (IMEB INC., San Marcos, CA), and leukocyte differentials were determined as percentage of 500 counted leukocytes.

The left lung lobe from infected mice was homogenized and subjected to Mp culture as previously reported [34]. The left lung from non-infected mice was fixed in 10% phosphate-buffered formalin and embedded in paraffin. Lung tissue block was cut at 5-µm thickness for immunohistochemical staining of SPLUNC1 as previously reported [35].

### Lung NF-κB Activity Assay

Mouse right lung tissues were homogenized and lyzed in nuclear protein extraction buffer to extract nuclear proteins as per manufacturer’s instruction (Active Motif, Carlsbad, CA). The ELISA-based chemiluminescent NF-κB activity assay was performed in the provided 96-well plate with immobilized multiple copies of NF-κB specific double-stranded oligonucleotide [36]. Briefly, equal amounts of nuclear proteins (20 µg) from each sample were loaded onto a 96-stripwell plate for binding to NF-κB consensus sequence. Samples and controls were incubated for one hour at room temperature with mild agitation to ensure successful binding to NF-κB consensus sequence. NF-κB binding was quantified with incubation of primary NF-κB antibody specific to the activated form of p65 subunit. Wells were then incubated with a horseradish peroxidase-conjugated antibody, and then a developing solution to provide an easily quantified, sensitive colorimetric readout. The absorbance was measured by spectrophotometer at 450 nm with reference to 655 nm wavelength.

### SPLUNC1 Immunohistochemistry (IHC)

Because there is no mouse SPLUNC1 ELISA available, we measured airway epithelial SPLUNC1 protein by IHC. Formalin-fixed and paraffin-embedded mouse lung sections were deparaffinized, rehydrated, followed by antigen retrieval with microwave boiling in 10 mM citrate buffer (pH 6.0) for 12 min. Sections were treated with 0.3% hydrogen peroxide in 0.05 M Tris buffered saline (TBS, pH 7.6) for 30 min to inhibit endogenous peroxidase, followed by incubation with 10% normal rabbit serum (Vector Laboratories, Burlingame, CA) for 30 min to block potential nonspecific binding sites. Then, the slides were incubated with a goat anti-mouse SPLUNC1 antibody (R&D systems, Minneapolis, MN) overnight at 4°C, followed by incubation with biotinylated horse anti-mouse IgG for one hour at room temperature. Thereafter, avidin-biotin-peroxidase complex (Vector Laboratories, Burlingame, CA) was added to the slides for 45 min at room temperature. After rinsing the slides in TBS, 0.03% aminoethylcarbazole (AEC) in 0.03% hydrogen peroxide was used as a substrate to develop a peroxide-dependent red color reaction. Nuclear counterstaining was performed by using Mayer’s hemotoxylin (Sigma-Aldrich, St. Louis, MO). The area of SPLUNC1 protein in epithelium of all medium-sized airways (defined as the basement membrane perimeter of 600–900 µm, and maximal diameter/minimal diameter ≤2, [26]) (n = 4 to 6 airways/mouse) lungs was quantified in a blinded fashion by using the National Institutes of Health Scion Image program (Bethesda, MD). The results were expressed as percentage of airway SPLUNC1 protein area/total airway epithelial area.

### ELISA of Mouse KC and IL-6

KC and IL-6 protein levels in mouse BALF were determined by using mouse KC and IL-6 DuoSet ELISA Development kits (R&D Systems, Minneapolis, MN) as per manufacturer’s instruction.

### Statistical Analysis

Normally distributed data were presented as means ± SEM and analyzed using the student’s *t*-test for two group comparison or two-way analysis of variance (ANOVA) for multiple group comparisons. Non-normally distributed data were compared using Wilcoxon rank-sum test. A value of P<0.05 was regarded as statistically significant.
